# Supplementary material for: Phylogeographic analysis of long-legged bats, Macrophyllum macrophyllum, with notes on roosting behavior and natural history
Source: PeerJ. 2025 May 23;13:e19432. doi: 10.7717/peerj.19432 (PMC12105620; doi:10.7717/peerj.19432)
Supplement: Supplemental Information 1 [file peerj-13-19432-s001.docx]

**Table S1:**

**Localities of *Macrophyllum macrophyllum* gleaned from the literature and used to produce the map in Figure 1.**

| **Locality** | **State/Department** | **Country** | **Coordinates** | **Decimal Latitude** | **Decimal longitude** | **Source** |
| --- | --- | --- | --- | --- | --- | --- |
| San Ignacio | Misiones | Argentina | 27°16'S, 55°32'W | -27.2667 | -55.5333 | Fornes, Delpietro & Massoia (1969) |
| Cita Ranch | Cayo | Belize | ca. 17°12'N, 88°35'W | 17.2 | -88.5833 | FMNH_database |
| Campamento El Trapiche | Beni | Bolivia | 14°48'S, 66°19'W | -14.8 | -66.3167 | Anderson (1997) |
| río Baures, mouth | Beni | Bolivia | 12°30'S, 64°18'W | -12.5 | -64.3 | Anderson (1997) |
| Tumichucua | Beni | Bolivia | 11°13'S, 66°14'W | -11.2167 | -66.2333 | Anderson (1997) |
| Centro Dieciocho | Pando | Bolivia | 10°36'S, 66°47'W | -10.6 | -66.7833 | Anderson (1997) |
| Independencia | Pando | Bolivia | 11°26'S, 67°34'W | -11.4333 | -67.5667 | Anderson (1997) |
| Santa Rosa | Pando | Bolivia | 12°13'S, 68°24'W | -12.2167 | -68.4 | Anderson (1997) |
| Río Negrillo | Santa Cruz | Bolivia | 15°03'S, 62°45'W | -15.05 | -62.75 | Anderson (1997) |
| Igarapé Três Bocas, Porto Walter | Acre | Brazil | ca. 08°16'S, 72°44'W | -8.26667 | -72.7333 | Nogueira, Pol & Peracchi (1999) |
| Maracá | Amapá | Brazil | ca. 00°26'S, 51°29'W | -0.43333 | -51.4833 | Castro & Michalski (2015) |
| Reserva de Desenvolvimento Sustentável do Rio Iratapuru | Amapá | Brazil | 0°16'37''N, 53°06'26''W | 0.260278 | -53.1072 | Martins, Bernard & Gregorin (2006) |
| Rodovia Perimetral Norte, km 160 | Amapá | Brazil | ca. 00°29'N, 50°36'W | 0.483333 | -50.6 | Peracchi, Raimundo & Tannure (1984) |
| Barcelos and Santa Isabel do Rio Negro | Amazonas | Brazil | ca. 00°37'S, 63°31'W | -0.61667 | -63.5167 | Moratelli, Dias & Bonvicino (2010) |
| João Pessoa (= Eirunepé) | Amazonas | Brazil | 06°39'S, 69°51'W | -6.66667 | -69.8667 | Patterson (1992) |
| Manaus | Amazonas | Brazil | ca. 03°04'S, 60°00'W | -3.06667 | -60 | Reis (1984) |
| Parque Nacional Jaú | Amazonas | Brazil | 01°54'S, 61°27'W | -1.9 | -61.45 | Barnett et al. (2006) |
| Tefé | Amazonas | Brazil | 03°22'S, 64°43'W | -3.36667 | -64.7167 | Mok & Lacey (1980) |
| Bahia (= Salvador) | Bahia | Brazil | ca. 13°00'S, 38°31'W | -13 | -38.5167 | Gervais (1856) |
| Lençois | Bahia | Brazil | 12°33'47''S, 41°24'05''W | -12.5631 | -41.4014 | Sbragia et al. (2010) |
| Paripiranga | Bahia | Brazil | 10°32’38”S, 37°57’23”W | -10.5439 | -37.9564 | Feijó et al. (2015) |
| Rio Mucuri | Bahia | Brazil | 18°05'S, 39°34'W | -18.0833 | -39.5667 | Wied (1826) coordinates from Vanzolini & Myers (2015) |
| Santa Luzia | Bahia | Brazil | 15°25'46''S, 39°20'03''W | -15.4294 | -39.3342 | Faria, Soares-Santos & Sampaio (2006) |
| Valença | Bahia | Brazil | ca. 13°22'S, 39°04'W | -13.3667 | -39.0667 | Faria, Soares-Santos & Sampaio (2006) |
| Morro d'Anta, Conceição da Barra | Espírito Santo | Brazil | 18°17'59"S, 39°57'11"W | -18.2997 | -39.9531 | Ruschi (1952) |
| Mucurici | Espírito Santo | Brazil | 18°05'S, 40°30'W | -18.0833 | -40.5 | Ruschi (1953) |
| São Mateus | Espírito Santo | Brazil | 18°43'S, 39°51'W | -18.7167 | -39.85 | Ruschi (1953) |
| Niquelândia (Fazenda Moinho) | Goiás | Brazil | ca. 14°29'S, 48°37'W | -14.4833 | -48.6167 | Coimbra et al. (1982) |
| Serra da Mesa | Goiás | Brazil | Not mapped | N/A | N/A | Fracasso & Salles (2005) |
| Uruaçu | Goiás | Brazil | ca. 14°31'S, 49°08'W | -14.5167 | -49.1333 | Triveiler (1998) |
| São Luís | Maranhão | Brazil | ca. 02°43'S, 44°16'W | -2.733 | -44.28 | Dias et al. (2007) |
| Parque Nacional da Serra da Bodoquena | Mato Grosso do Sul | Brazil | 20°40'S, 56°46'W | -20.67 | -56.78 | Camargo et al. (2009) |
| Caratinga | Minas Gerais | Brazil | 19°47'S, 42°08'W | -19.7833 | -42.1333 | Taddei (1975) |
| Ipatinga | Minas Gerais | Brazil | 19°28'S, 42°32'W | -19.4667 | -42.5333 | Vieira (1942) |
| Parque Estadual do Rio Doce, Marliéria | Minas Gerais | Brazil | ca. 19°42’S, 42°34’W | -19.7 | -42.5667 | Tavares & Anciães (1998) |
| Uberlândia | Minas Gerais | Brazil | ca. 18°55'S, 48°16'W | -18.9 | -48.28 | Stutz et al. (2004) |
| Belém, Utinga | Pará | Brazil | 01°27'S 48°29'W | -1.45 | -48.4833 | USNM 361504 |
| Floresta Nacional de Caxiuanã | Pará | Brazil | ca. 01°47'S, 51°26'W | -1.45 | -51.4333 | Marques-Aguiar & Aguiar (2002) |
| Ilha do Taiuna (= Ilha do Tayaúna) | Pará | Brazil | 02°15'S, 49°29'W | -2.25 | -49.4833 | Harrison (1975) |
| Rio Tapajós, Parque Nacional da Amazônia | Pará | Brazil | 04°34'S, 56°18'W | -4.56667 | -56.3 | Reis & Schubart (1979) |
| Rio Xingu, 52 km SSW Altamira | Pará | Brazil | 03°39'S, 52°22'W | -3.65 | -52.3667 | Voss & Emmons (1996) |
| Sapé | Paraíba | Brazil | ca. 07°05'S, 35°13'W | -7.08333 | -35.2167 | Feijó & Langguth (2011) |
| Candói | Paraná | Brazil | 25°39'S, 51°57'W | -25.65 | -51.95 | Miranda et al. (2019) |
| Sumidouro, Rio Branco do Sul | Paraná | Brazil | ca. 25°11'S 49°18'W | -25.1833 | -49.3 | Miretzki (2003) |
| Estação Ecológica do Tapacurá, São Lourenço da Mata | Pernambuco | Brazil | ca. 08°00'S, 35°01'W | -8 | -35.0167 | Feijó & Langguth (2011) |
| Ilha da Gipóia, Angra dos Reis | Rio de Janeiro | Brazil | 23°02'S 44°01'W | -23.0333 | -44.0167 | Carvalho et al. (2011) |
| Itaperuna | Rio de Janeiro | Brazil | 21°12'S 41°53'W | -21.2 | -41.8833 | Louzada, Nogueira & Pessoa (2021) |
| Rio das Pedras, Mangaratiba | Rio de Janeiro | Brazil | 22°59'S, 44°05'W | -22.9928 | -44.095 | Muylaert et al. (2017) |
| Sumidouro | Rio de Janeiro | Brazil | 22°07'38"S, 42°41'00"W | -22.1272 | -42.6833 | Novaes et al. (2015) |
| Universidade Federal Rural do Rio de Janeiro, Seropédica | Rio de Janeiro | Brazil | 22°45'S, 43°41' | -22.75 | -43.6833 | Peracchi & Albuquerque (1971) |
| Vala da Banana, APA Guapimirim | Rio de Janeiro | Brazil | 22°41'S, 42°59'W | -22.6868 | -42.996 | Louzada, Nogueira & Pessoa (2021) |
| Cachoeira de Emas | São Paulo | Brazil | 21°55'S 47°21'W | -21.9167 | -47.35 | Vieira (1955) |
| Icém | São Paulo | Brazil | 20°21'S 49°16'W | -20.35 | -49.2667 | Taddei (1975) |
| Onda Branca, 10 km NW Nova Granada | São Paulo | Brazil | 20°27'S 49°23'W | -20.45 | -49.3833 | Taddei (1975) |
| Parque Estadual Turistico do Alto Ribeira, Iporanga | São Paulo | Brazil | 24°31'S 48°42'W | -24.5167 | -48.7 | Garbino (2016) |
| Sales | São Paulo | Brazil | 21°20'S 49°29'W | -21.3333 | -49.4833 | Taddei (1975) |
| Campus Universidade Federal de Sergipe, São Cristóvão | Sergipe | Brazil | 10°55'S, 37°04'W | -10.9167 | -37.0667 | Rocha et al. (2010) |
| Leticia | Amazonas | Colombia | 04°09'S, 69°57'W | -4.15 | -69.95 | USNM 595074 |
| Chigorodó | Antioquia | Colombia | 07°41'N, 76°42'W | 7.683333 | -76.7 | USNM 431758 |
| Bonda | Magdalena | Colombia | 11°14'N, 74°07'W | 11.23333 | -74.1167 | Allen (1900) |
| Acacías | Meta | Colombia | 03°59'N 73°45'W | 3.983333 | -73.75 | USNM 595075 |
| Buenaventura | Valle del Cauca | Colombia | 03°56'N, 77°04'W | 3.933333 | -77.0667 | Williams & Genoways (2008) |
| Caño Cubiyu (= Cubigu), Rio Vaupes, 50 Km W Mitu | Vaupés | Colombia | ca. 01°02'N, 70°12'W | 1.033333 | -70.2 | USNM 483328 |
| La Pacifica, COMELCO | Guanacaste | Costa Rica | ca. 10°15'N 85°36'W | 10.25 | -85.6 | LaVal & Fitch (1977) |
| Río Colorado, 9 km N Liberia | Guanacaste | Costa Rica | 10°38'N 85°33'W | 10.63333 | -85.55 | Starrett & Casebeer (1968) |
| La Selva Biological Station | Heredia | Costa Rica | 10°26'N 83°59'W | 10.43333 | -83.9833 | LaVal & Fitch (1977) |
| Finca La Lola, Río Madre de Dios | Limón | Costa Rica | 10°05'N, 83°23'W | 10.08333 | -83.3833 | Starrett & Casebeer (1968) |
| Estero Inés | Esmeraldas | Ecuador | 00°41’N 80°02’W | 0.683333 | -80.0333 | Tirira & Boada (2005) |
| San Francisco de Bogotá | Esmeraldas | Ecuador | 01°02’N 78°25’W | 1.033333 | -78.4167 | Carrera et al. (2010) |
| Laguna Grande, Río Cuyabeno | Napo | Ecuador | 00°00' 76°11'W | 0 | -76.1833 | Albuja (1999) |
| Parque Nacional Yasuni | Napo | Ecuador | 00°40'S 76°25'W | -0.68 | -76.43 | Clare et al. (2011) |
| Putsu | Pastaza | Ecuador | 01°50'S 77°50'W | -1.83333 | -77.8333 | Hill & Bown (1963) |
| Limoncocha | Sucumbíos | Ecuador | 00°24'S, 76°36'W | -0.4 | -76.6 | Albuja (1999) |
| Marian | Sucumbíos | Ecuador | 00°01'S, 76°20'W | -0.01667 | -76.3333 | Albuja (1999) |
| Cueva Hedionda | La Libertad | El Salvador | 13°31'N 89°19'W | 13.51667 | -89.3167 | Hill & Bown (1963) |
| Acajutla |  | El Salvador | 13°35'N 89°49'W | 13.58333 | -89.8167 | Harrison & Pendleton (1974) |
| Paracou, near Sinnamary |  | French Guiana | 05°17'N, 52°55'W | 5.283333 | -52.9167 | Simmons & Voss (1998) |
| Sinnamary |  | French Guiana | 05°23'N, 52°57'W | 5.383333 | -52.95 | Brosset & Dubost (1967) |
| La Avellana |  | Guatemala | 13°55'N 90°28'W | 13.91667 | -90.4667 | Seymour & Dickerman (1982) |
| 24 miles from Bartica on Potaro Road | Cuyuni-Mazaruni | Guyana | 06°00'N, 58°40'W | 6 | -58.6667 | Hill (1964) |
| Río Sicre (= Río Sigre) | Gracias a Dios | Honduras | ca. 15°45'N, 84°35'W | 15.75 | -84.5833 | Handley (1957) |
| Río Coco, 76 miles E Danlí |  | Honduras | ca. 14°05'N, 85°38'W | 14.08333 | -85.6333 | Davis, Carter & Pine (1964) |
| Arroyo José | Chiapas | Mexico | 16°06'N, 91°56'W | 16.1 | -91.9333 | Alvarez-Castañeda & Alvarez (1991) |
| Arroyo San Pablo | Chiapas | Mexico | 16°05'N, 91°57'W | 16.08333 | -91.95 | Alvarez-Castañeda & Alvarez (1991) |
| Estación Chajul de SEDUE | Chiapas | Mexico | 16°06'N, 90°56'W | 16.1 | -90.9333 | Alvarez-Castañeda & Alvarez (1991) |
| Río Lagartos | Chiapas | Mexico | 16°07'N, 91°57'W | 16.11667 | -91.95 | Alvarez-Castañeda & Alvarez (1991) |
| Teapa | Tabasco | Mexico | 17°32'N, 92°45'W | 17.53333 | -92.75 | Lay (1962) |
| Cacao (2 km W from Muelle de los Bueyes) | Región Autónoma de la Costa Caribe Sur | Nicaragua | ca. 12°03'N, 84°32'W | 12.05 | -84.5333 | Davis et al. (1964) |
| 12 km SSW Changuinola | Bocas del Toro | Panama | 09°27'N, 82°31'W | 9.45 | -82.5167 | Handley (1966) |
| Almirante | Bocas del Toro | Panama | 09°18'N, 82°24'W | 9.3 | -82.4 | Handley (1966) |
| Fort Davis | Colón | Panama | 09°15'N, 79°56'W | 9.25 | -79.9333 | Handley (1966) |
| Fort Gulick | Colón | Panama | 09°18'N, 79°53'W | 9.3 | -79.8833 | Handley (1966) |
| Madden Dam | Colón | Panama | 09°13'N, 79°38'W | 9.216667 | -79.6333 | Handley (1966) |
| Salamanca Hydrographic Station | Colón | Panama | 09°17'N, 79°36'W | 9.283333 | -79.6 | Handley (1966) |
| Boca del Rio Paya | Darién | Panama | 07°55'N, 77°31'W | 7.916667 | -77.5167 | Handley (1966) |
| Jaqué | Darién | Panama | 07°31'N, 78°10'W | 7.516667 | -78.1667 | Handley (1966) |
| Armila | Guna Yala | Panama | 08°40'N, 77°27'W | 8.666667 | -77.45 | Handley (1966) |
| Puerto Obaldía | Guna Yala | Panama | 08°40'N, 77°25'W | 8.666667 | -77.4167 | Handley (1966) |
| Cerro Azul | Panamá | Panama | 09°13'N, 79°18'W | 9.216667 | -79.3 | Handley (1966) |
| Chepo | Panamá | Panama | 09°10'N, 79°06'W | 9.166667 | -79.1 | Handley (1966) |
| Pacora | Panamá | Panama | 09°04'N, 79°18'W | 9.066667 | -79.3 | Handley (1966) |
| Panama city | Panamá | Panama | 08°59'N, 79°31'W |  |  | Goldman (1920) |
| Panama Viejo | Panamá | Panama | 08°59'N, 79°31'W | 8.983333 | -79.5167 | Handley (1966) |
| 2 miles S San Francisco | Veraguas | Panama |  |  |  | Davis et al. (1964) |
| Cerro Hoya | Veraguas | Panama | 07°18'N, 80°42'W | 7.3 | -80.7 | Handley (1966) |
| Arroyo Tacuara | Amambay | Paraguay | 22°30'S, 56°00'W | -22.5 | -56 | Wilson & Gamarra de Fox (1991) |
| Pedro Juan Caballero, Cerro Cora | Amambay | Paraguay | 22°37'S, 56°01'W | -22.63 | -56.02 | Muyalert et al. (2017) |
| Rio Ypané, Belén | Concepción | Paraguay | 23°27'S, 57°15'W | -23.45 | -57.25 | Baud (1989) |
| Río Cenipa, mouth | Amazonas | Peru | 04°40'S, 78°12'W | -4.66667 | -78.2 | Tuttle (1970) |
| Nuevo San Juan | Loreto | Peru | 05°15'S, 73°10'W | -5.25 | -73.1667 | Voss et al. (2016) |
| San Jeronimo, rio Ucayali | Loreto | Peru | 07°55'S, 74°54'W | -7.92 | -74.91 | Thomas (1928), coordinates from Patton, Pardiñas & D’Elía (2015) |
| Pakitza | Madre de Dios | Peru | 11°55'S, 71°15'W | -11.9167 | -71.25 | Ascorra et al. (1991) |
| San Juan | Pasco | Peru | 10°30'S, 74°53'W | -10.5 | -74.8833 | Tuttle (1970) |
| Balta | Ucayali | Peru | 10°08'S, 71°13'W | -10.1333 | -71.2167 | Voss & Emmons (1996) |
| Coronel Portillo, Yarinacocha | Ucayali | Peru | 08°17'S, 74°39'W | -8.28333 | -74.65 | Sanborn (1949) |
| Santo Boma Locks | Wanica | Suriname | 05°47'N, 55°17'W | 5.783333 | -55.2833 | Husson (1978) |
| Belén, Río Cunucunuma | Amazonas | Venezuela | 03°39'N, 65°46'W | 3.65 | -65.7667 | Handley (1976) |
| Boca Mavaca | Amazonas | Venezuela | 02°30'N, 65°13'W | 2.5 | -65.2167 | Handley (1976) |
| Capibara, Brazo Casiquiare | Amazonas | Venezuela | 02°38'N, 66°19'W | 2.633333 | -66.3167 | Handley (1976) |
| El Merey | Amazonas | Venezuela | ca. 03°05'N, 65°55'W | 3.083333 | -65.9167 | Handley (1976) |
| La Esmeralda | Amazonas | Venezuela | 03°10'N, 65°33'W | 3.166667 | -65.55 | Handley (1976) |
| Río Mavaca, 108 km SSE Esmeralda | Amazonas | Venezuela | 02°15'N, 65°17'W | 2.25 | -65.2833 | Handley (1976) |
| San Juan, Rio Manapiare | Amazonas | Venezuela | 05°18'N, 66°13'W | 5.3 | -66.2167 | Handley (1976) |
| Hato Caribén, Río Cinaruco, 32 to 46 km NE Pto. Paez | Apure | Venezuela | 06°33'N, 67°13'W | 6.55 | -67.2167 | Handley (1976) |
| Laguna de Macupino (= Laguna Macupina), Santa María | Apure | Venezuela | 06°27'N, 67°12'W | 6.46 | -67.2 | Linares (1966) |
| El Manaco, 59 km SE El Dorado | Bolívar | Venezuela | 06°21'N, 61°17'W | 6.35 | -61.2833 | Handley (1976) |
| Río Supamo, 50 km SE El Manteco | Bolívar | Venezuela | 07°00'N, 62°15'W | 7 | -62.25 | Handley (1976) |
| Lago de Valencia | Carabobo | Venezuela | 10°11'N, 67°45'W | 10.18333 | -67.75 | Linares (1966) |
| Caño Araguabisi | Delta Amacuro | Venezuela | 09°29'N, 60°57'W | 9.483333 | -60.95 | Linares & Rivas (2004) |
| Embalse de Guárico | Guárico | Venezuela | 09°01'N, 67°26'W | 9.016667 | -67.4333 | Handley (1976) |
| Hato Las Palmitas, 35 km SSW San Juan de los Morros | Guárico | Venezuela | 09°36'N, 67°27'W | 9.6 | -67.45 | Handley (1976) |
| Cueva de Saffont, Caripe | Monagas | Venezuela | ca. 10°10'N, 63°32'W | 10.16667 | -63.5333 | Linares (1966) |
| El Rosario, 65 kmWNW of Los Encontrados | Zulia | Venezuela | ca. 09°18'N, 72°46'W | 9.3 | -72.7667 | Handley (1976) |
| Lagunillas | Zulia | Venezuela | 10°08°N, 71°16'W | 10.13333 | -71.2667 | Handley (1956) |

**References**

Albuja L. 1999. *Murciélagos del Ecuador*. Quito: Cicetrónic Cía. Ltda. Offset.

Allen JA. 1900. List of bats collected by Mr. H. H. Smith in the Santa Marta region of Colombia, with descriptions of new species. *Bulletin of the American Museum of Natural History* 13:87–94.

Alvarez-Castañeda ST, Alvarez T. 1991. *Los Murciélagos de Chiapas*. México, D.F.: Instituto Politecnico Nacional.

Anderson S. 1997. Mammals of Bolivia, taxonomy and distribution. *Bulletin of the American Museum of Natural History* 231:1–652. DOI: 10.1080/00222938800770331.

Barnett AA, Sampaio EM, Kalko EK V., Shapley RL, Fischer E, Camargo G, Rodríguez-Herrera B. 2006. Bats of Jaú National Park, central Amazônia, Brazil. *Acta Chiropterologica* 8:103–128.

Baud FJ. 1989. Présence de *Macrophyllum macrophyllum* Schinz (Chiroptera, Phyllostominae) au Paraguay. *Mammalia* 53:308–309.

Brosset A, Dubost G. 1967. Chiropteres de la Guyane Française. *Mammalia* 31:583–594.

Camargo G, Fischer E, Gonçalves F, Fernandes G, Ferreira S. 2009. Morcegos do Parque Nacional da Serra da Bodoquena, Mato Grosso do Sul, Brasil. *Chiroptera Neotropical* 15:417–424.

Carvalho WD, Freitas LN, Freitas GP, Luz JL, Costa LM, Esbérard CEL. 2011. Efeito da chuva na captura de morcegos em uma ilha da costa sul do Rio de Janeiro, Brasil. *Chiroptera Neotropical* 17:808–816.

Castro IJ, Michalski F. 2015. Bats of a varzea forest in the estuary of the Amazon River, state of Amapá, Northern Brazil. *Biota Neotropica* 15:1–8. DOI: 10.1590/1676-06032015016814.

Clare EL, Lim BK, Fenton MB, Hebert PDN. 2011. Neotropical Bats: Estimating Species Diversity with DNA Barcodes. *PLOS ONE* 6:1–14. DOI: 10.1371/journal.pone.0022648.

Coimbra CEA, Borges MM, Guerra DQ, Mello DA. 1982. Contribuição à zoogeografia e ecologia de morcegos em regiões de cerrado do Brasil Central. *Boletim Técnico do IBDF* 7:34–38.

Davis WB, Carter DC, Pine RH. 1964. Noteworthy Records of Mexican and Central American Bats. *Journal of Mammalogy* 45:287–375. DOI: 10.2307/1377410.

Dias PA, Santos CLC, Rodrigues FS, Rosa LC, Lobato KS, Rebêlo JMM. 2007. Espécies de moscas ectoparasitas (Diptera, Hippoboscoidea) de morcegos (Mammalia, Chiroptera) no estado do Maranhão. *Revista Brasileira de Entomologia* 53:128–133.

Faria D, Soares-Santos B, Sampaio E. 2006. Bats from the Atlantic rainforest of southern Bahia, Brazil. *Biota Neotropica* 6:0–0. DOI: 10.1590/S1676-06032006000200022.

Feijó JA, Langguth A. 2011. Lista de Quirópteros da Paraíba, Brasil com 25 novos registros. *Chiroptera Neotropical* 17:1055–1062.

Feijó A, Rocha PA, Mikalauskas J, Ferrari SF. 2015. *Macrophyllum macrophyllum* (Chiroptera, Phyllostomidae) in the Brazilian Caatinga scrublands: rivers basins as potential routes of dispersal in xeric ecosystems. *Mastozoología Neotropical* 22:163–169.

Fornes A, Delpietro H, Massoia E. 1969. *Macrophyllum macrophyllum* (Wied) nuevo genero y especie para la Republica Argentina (Chiroptera, Phyllostomidae, Phyllostominae). *Physis* 28:323–326.

Fracasso MP de A, Salles LO. 2005. Diversity of quaternary bats from Serra da Mesa (State of Goiás, Brazil). *Zootaxa* 817:1–19.

Garbino GST. 2016. Research on bats (Chiroptera) from the state of São Paulo, Southeastern Brazil: annotated species list and bibliographic review. *Arquivos de Zoologia, Museu de Zoologia da Universidade de São Paulo* 47:43–128.

Gervais P. 1856. Deuxième mémoire. Documents zoologiques pour servir à la monographie des Chéiroptères sud-américains. In: Castelnau F de ed. *Animaux nouveaux ou rares recueillis pendant l’expédition dans les parties centrales de l’Amérique du Sud, de Rio de Janeiro à Lima, et de Lima au Para*. Paris: P. Bertrand, 25–88.

Goldman EA. 1920. Mammals of Panama. *Smithsonian Miscellaneous Collections* 69:1–309.

Handley CO. 1957. Firest records of the occurrence of the long-legged bat (*Macrophyllum*) in Honduras and Venezuela. *Journal of Mammalogy* 38:406–407.

Handley CO. 1966. Checklist of the mammals of Panama. In: Wenzel RL, Tipton VJ eds. *Ectoparasites of Panama*. Chicago, IL: Field Musem of Natural History, 753–795.

Handley CO. 1976. Mammals of the Smithsonian Venezuelan Project. *Brigham Young University Science Bulletin, Biological Series* 20:1–89. DOI: http://scholarsarchive.byu.edu/byuscib/vol20/iss5/1/.

Harrison DL. 1975. Macrophyllum macrophyllum. *Mammalian Species* 62:1–3.

Harrison DL, Pendleton N. 1974. A second recod of Wied’s long-legged bat *Macrophyllum macrophyllum* (Chiroptera; Phyllostomatidae) in El Salvador, with notes on the palate, reproduction and diet of the species. *Mammalia* 38:689–693.

Hill JE. 1964. Notes on bats from British Guiana, with the description of a new genus and species of *Phyllostomidae*. *Mammalia* 28:553–572.

Hill JE, Bown A. 1963. Occurrence of *Macrophyllum* in Ecuador. *Journal of Mammalogy* 44:588.

Husson AM. 1978. *The Mammals of Suriname*. Leiden: E. J. Brill.

LaVal RK, Fitch HS. 1977. Structure, movements and reproduction in three Costa Rican bat communities. *Occasional Papers, Museum of Natural History of the University of Kansas* 69:1.

Lay DM. 1962. Seis mamiferos nuevos para la fauna de Mexico. *Anales del Instituto de Biología* 33:373–377.

Linares OJ. 1966. Notas acerca de *Macrophyllum macrophyllum* (Wied) (Chiroptera). *Memoria de la Sociedad de Ciencias Naturales La Salle* 26:53–61.

Linares OJ, Rivas AB. 2004. Mamíferos del Sistema Deltaico (dental del Orinoco-golfo de Paria), Venezuela. *Memoria de la Fundación La Salle de Ciencias Naturales* 159–160:27–104.

Louzada NS V., Nogueira M, Pessoa LM. 2021. First record of *Macrophyllum macrophyllum* (Schinz, 1821) in Brazilian mangroves, with comments on bat diversity in this ecosystem. *Notas sobre Mamíferos Sudamericanos* 03:001–013. DOI: 10.31687/saremnms.21.6.2.

Marques-Aguiar SA, Aguiar GFS. 2002. Interações de quirópteros em ecossistemas tropicais: perspectivas de estudo para Caxiuanã. In: Lisboa PLB ed. *Caxiuanã: Populações tradicionais, meio físico e diversidade biológica*. Belém: Museu Paraense Emílio Goeldi, 651–668.

Martins ACM, Bernard E, Gregorin R. 2006. Inventários biológicos rápidos de morcegos (Mammalia, Chiroptera) em três unidades de conservação do Amapá, Brasil. *Revista Brasileira de Zoologia*:1175–1184.

Miranda JMD, da Silva LZ, Pressinatte-Júnior S, Pereira L de A, Marchioro S, Bôlla DAS, Carvalho F. 2019. Bat fauna (Mammalia, chiroptera) from guarapuava highlands, Southern Brazil. *Oecologia Australis* 23:562–574. DOI: 10.4257/oeco.2019.2303.14.

Miretzki M. 2003. Morcegos do estado do Paraná, Brasil (Mammalia, Chiroptera): riqueza de espécies, distribuição e síntese do conhecimento atual. *Papéis Avulsos de Zoologia (São Paulo)* 43:101–138. DOI: 10.1590/S0031-10492003000600001.

Mok WY, Lacey LA. 1980. Algumas considerações ecológicas sobre morcegos vampiros na epidemiologia da raiva humana na Bacia Amazônica. *Acta Amazonica* 10:335–342.

Moratelli R, Dias D, Bonvicino CR. 2010. Estrutura e análise zoogeográfica de uma taxocenose de morcegos no norte do Estado do Amazonas, Brasil. *Chiroptera Neotropical* 16:661–671.

Muylaert RDL, Stevens RD, Esbérard CEL, Mello MAR, Garbino GST, Varzinczak LH, Faria D, Weber MDM, Kerches Rogeri P, Regolin AL, Oliveira HFMD, Costa LDM, Barros MAS, Sabino-Santos G, Crepaldi de Morais MA, Kavagutti VS, Passos FC, Marjakangas E-L, Maia FGM, Ribeiro MC, Galetti M. 2017. ATLANTIC BATS: a data set of bat communities from the Atlantic Forests of South America. *Ecology* 98. DOI: 10.1002/ecy.2007.

Nogueira MR, Pol A, Peracchi AL. 1999. New records of bats from Brazil with a list of additional species for the chiropteran fauna of the state of Acre, western Amazon basin. *Mammalia* 63:363–368.

Novaes RLM, Rosa DTC, Vrcibradic D, Avilla L dos S. 2015. Bat assemblages from three Atlantic Forest fragments in Rio de Janeiro state, Southeastern Brazil. *Biodiversity Data Journal* 3:e4404. DOI: 10.3897/BDJ.3.e4404.

Patterson BD. 1992. Mammals in the Royal Natural History Museum, Stockholm, Collected in Brazil and Bolivia by A. M. Olalla during 1934-1938. *Fieldiana Zoology, New Series* 66:1–64.

Patton JL, Pardiñas U, D’Elía G. 2015. *Mammals of South America, Volume 2: Rodents*. Chicago: The University of Chicago Press.

Peracchi AL, Albuquerque ST. 1971. Lista provisória dos quirópteros dos Estados do Rio de Janeiro e Guanabara, Brasil (Mammalia, Chiroptera). *Revista Brasileira de Biologia* 31:405–413.

Peracchi AL, Raimundo SDL, Tannure AM. 1984. Quirópteros do Território Federal do Amapá, Brasil (Mammalia, Chiroptera). *Arquivos da Universidade Federal Rural do Rio de Janeiro* 7:89–100.

Reis NR. 1984. Estrutura de comunidade de morcegos na região de Manaus, Amazonas. *Revista Brasileira de Biologia* 44:247–254.

Reis NR, Schubart HOR. 1979. Notas preliminares sobre os morcegos do Parque Nacional da Amazônia (Médio Tapajós). *Acta Amazonica* 9:507–515.

Rocha PA, Mikalauskas JS, Gouveia SF, Silveira VV-B, Peracchi AL. 2010. Morcegos (Mammalia, Chiroptera) capturados no Campus da Universidade Federal de Sergipe, com oito novos registros para o estado. *Biota Neotroprica* 10:183–188.

Ruschi A. 1952. Morcegos do Estado do Espírito Santo IX. Os morcegos das grutas do Limoeiro em Castelo, Monte Líbano em Cachoeiro do Itapemirim e de Itaúnas, em Morro d’Anta, em Conceição da Barra. Grutas de Inverno, Verão e Acidentais. Cohabitação. O banho, Morcegário e criação em cativeiro. Pesquisas sôbre Corpusculos de Negri. *Boletim do Museu de Biologia Prof. Mello-Leitão* 9:1–88.

Ruschi A. 1953. Morcegos do Estado do Espírito Santo XIV. Família Phyllostomidae. Descrição das espécies: *Dolichophillum macrophyllum* (Wied) e *Chrotopterus auritus australis* (Thomas). *Boletim do Museu de Biologia Prof. Mello-Leitão* 16:1–11.

Sanborn CC. 1949. Mammals from the Rio Ucayali, Peru. *Journal of Mammalogy* 30:277–288.

Sbragia IA, Corrêa MM de O, Pessôa LM, Oliveira J. 2010. The karyotype of *Macrophyllum macrophyllum* (Schinz, 1821) (Phyllostomidae: Phyllostominae) from the state of Bahia, Brazil. *Chiroptera Neotropical* 16:600–602.

Seymour C, Dickerman RW. 1982. Observations of the long-legged bat, *Macrophyllum macrophyllum*, in Guatemala. *Journal of Mammalogy* 63:530–532.

Simmons NB, Voss RS. 1998. The Mammals of Paracou, French Guiana: a neotropical lowland rainforest fauna Part 1. Bats. *Bulletin of the American Museum of Natural History*:1–219.

Starrett A, Casebeer RS. 1968. Records of bats from Costa Rica. *Contributions in Science, Los Angeles County Museum* 148:1–21.

Stutz WH, Albuquerque MC, Uieda W, Macedo EM, França CB. 2004. Updated list of Uberlândia bats (Minas Gerais State, southeastern Brazil). *Chiroptera Neotropical* 10:188–190.

Taddei VA. 1975. Phyllostomidae (Chiroptera) do norte-ocidental do Estado de São Paulo. I – Phyllostominae. *Ciência e Cultura* 27:621–632.

Tavares VC, Anciães M. 1998. Artificial roosts and diet of some insectivorous bats in the Parque Estadual do Rio Doce, Brazil. *Bat Research News* 39:142.

Thomas O. 1928. The Godman-Thomas expedition to Peru.–VII. The mammals of the Rio Ucayali. *The Annals and Magazine of Natural History* 2:249–265.

Tirira D, Boada C. 2005. Evaluación ecológica rápida de la mastofauna en los bosques del suroccidente de la provincia de Esmeraldas. In: Vázquez MA, Freire JF, Suárez L eds. *Biodiversidad en el suroccidente de la provincia de Esmeraldas : Un reporte de las evaluaciones ecológicas y socioeconómicas rápidas*. Quito: EcoCiencia, 109–127.

Triveiler F. 1998. Estrutura e composição da fauna de quirópteros da região do alto Tocantins, GO. Porto Alegre: Universidade Federal do Rio Grande do Sul.

Tuttle MD. 1970. Distribution and zoogeography of Peruvian Bats, with comments on Natural History. *The University of Kansas Science Bulletin* 49:45–86.

Vanzolini PE, Myers CW. 2015. The Herpetological Collection of Maximilian, Prince of Wied (1782–1867), With Special Reference To Brazilian Materials. *Bulletin of the American Museum of Natural History* 2015:1–155. DOI: 10.1206/910.1.

Vieira CO da C. 1942. Ensaio monográfico sobre os quirópteros do Brasil. *Arquivos de Zoologia, Museu de Zoologia da Universidade de São Paulo* 3:219–471.

Vieira CO da C. 1955. Lista remissiva dos mamíferos do Brasil. *Arquivos de Zoologia, Museu de Zoologia da Universidade de São Paulo* 8:341–474.

Voss RS, Emmons LH. 1996. Mammalian Diversity in Neotropical Lowland Rainforests : a Preliminary Assessment. *Bulletin of the American Museum of Natural History* 230:1–115.

Voss RS, Fleck DW, Strauss RE, Velazco PM, Simmons NB. 2016. Roosting ecology of Amazonian bats: evidence for guild structure in hyperdiverse mammalian communities. *American Museum Novitates* 3870:1–43. DOI: 10.1206/3870.1.

Wied M. 1826. *Beiträge zur Naturgeschichte von Brasilien. II. Band*. Weimar: Landes-Industrie-Comptoir.

Williams SL, Genoways HH. 2008. Subfamily Phyllostominae Gray, 1825. In: Gardner AL ed. *Mammals of South America, Volume 1: marsupials, xenarthrans, shrews, and bats*. Chicago, IL, 255–300.

Wilson DE, Gamarra de Fox I. 1991. El murcielago *Macrophyllum macrophyllum* (Chiroptera: Phyllostomidae) en Paraguay. *Boletin de Museo Nacional de Historia Natural del Paraguay* 10:33–35.
